# Preparation and Self-Healing Properties of Clinker/PVP Microsphere in Cement Paste

**DOI:** 10.3390/ma13030589

**Published:** 2020-01-27

**Authors:** Jun Li, Zhengwu Jiang, Wenting Li

**Affiliations:** Key Laboratory of Advanced Civil Engineering Materials of Ministry of Education, School of Materials Science and Engineering, Tongji University, Shanghai 201804, China; 1610421@tongji.edu.cn (J.L.); lwt@tongji.edu.cn (W.L.)

**Keywords:** autolytic mineral self-healing, clinker, film coating, autolytic behavior

## Abstract

This paper presents a new insight into the autolytic mineral self-healing method for cementitious materials. The clinker/PVP (polyvinyl pyrrolidone) autolytic microsphere was prepared via the film coating method with cement clinker as a healing agent and PVP as the autolytic coating film. The morphology and chemical structure of the microsphere were characterized by environmental scanning electron microscopy (FESEM) equipped with energy dispersive spectrometer (EDS) and Fourier transform infrared spectroscopy (FTIR), respectively. The clinker retaining original mineral healing composition was successfully coated with a PVP film confirmed by FTIR. The maximum film thickness was 7.54 μm, which was determined by laser particle size measurement. The autolytic behavior was measured using isothermal calorimetry and successfully controlled by pretreatment degree (i.e., silane coupling agent amount). Experimental results showed that the compressive strength recovery of cement paste with a 30% microsphere was 54% higher than ordinary cement paste specimens. The damage degree of the specimen was also decreased by adding the autolytic microsphere.

## 1. Introduction

Concrete is the most widely used material in modern construction since Portland cement was developed in the 1820s. Concrete has outstanding properties in terms of high compressive strength, excellent molding performance and low cost. However, cracks in concrete structure occur easily because of its relatively low flexural strength compared to its high compressive strength [1,2]. The cracking of concrete structures is the most important factor causing or accelerating the major deteriorating processes of reinforced concrete, such as alkali-aggregate reactions, sulfate erosion and steel bar corrosion. Many hazardous substances permeate these cracks with water, especially those cracks which connect with each other, thus causing the deterioration of the concrete and shortening the structure service life [3,4]. Although man-made repair is a conventional solution, problems ae often encountered in repair work resulting from not-visible or inaccessible cracks, or structures in complex environments [5,6,7]. Moreover, it is high-cost and high-labor operation work which only lasts 10–15 years [8,9,10]; thus, there are calls for a more sustainable approach [11,12,13]. Considering this, crack self-healing would be more effective. The well-known autogenous self-healing is actually a natural phenomenon of cementitious materials [14]. Limited crack width is healed mainly due to the mechanism of the further hydration of unhydrated cement, or the carbonation of calcium hydroxide (Ca(OH)_2_). The younger samples present better self-healing effectiveness [15]. In addition, the healing efficiency of autogenous healing is poor and difficult to control [4].

Therefore, there are many healing methods which have been designed to enhance autogenous self-healing, including the addition of mineral admixtures, bacteria and adhesive agents [16,17,18]. Based on whether there is any host–guest reaction, mineral self-healing can be classified as intrinsic healing, which presents better compatibility [19,20,21]. Furthermore, the permeability and strength recovery of mineral self-healing are more effective [22,23,24]. However, there are several problems that challenge mineral self-healing, in which uncontrollable healing time is one of them. Therefore, inspired by the autolysis of biological cells, the autolytic mineral self-healing method has been proposed in previous groups’ research [25]. The autolytic mineral self-healing method is similar to the process of cell autolysis that refers to self-destruction by its enzyme. The autolytic microsphere used in this method consists of the internal healing substance and the external protective coating film. The healing substance can be a mineral material, such as cement clinker, or a monomineral that can produce healing products like C-S-H—which has a certain strength and great compatibility with the cementitious materials.

This paper presents the preparation and self-healing characterization of a clinker/PVP autolytic microsphere. The microsphere consists of cement clinker as a healing agent and polyvinyl pyrrolidone (PVP) as a film to avoid premature consumption [26]. Clinker is a suitable and compatible mineral since it is the main raw material of concrete. Moreover, PVP film can withstand the shear force due to its high tensile strength, so that coating film will not be broken in the mix procedure [27,28,29]. Silane coupling agent is chosen as the molecular bridge between the inorganic (i.e., clinker) and organic (i.e., PVP) matrix. Potential use of clinker as a self-healing constituent was investigated by a researcher. The possible self-healing phenomena were detected using hydration products and the recovery of strength, even though only a preliminary step was conducted [30].

In this study, the clinker was treated with silane coupling agent and then coated with an autolytic film. The morphology and chemical structure of the autolytic microsphere were determined. The film thickness was also measured. In addition, the self-healing effectiveness was evaluated via measurement of compressive strength recovery and a damage degree test.

## 2. Materials and Methods 

### 2.1. Materials

All reagents used in this experiment were produced from Sinopharm Chemical Reagent Co., Ltd. (Shanghai, China) Polyvinyl pyrrolidone (PVP) in analytic grade was chosen as the film material. Silane coupling agent (γ-aminopropyl triethoxysilane, KH-550) was used as a surface pretreatment agent for the cement clinker—the surface pretreatment agent which was chosen to form the active binding layer on the surface of clinker. The chemical composition of cement clinker was shown in Table 1.

### 2.2. Preparation of clinker/PVP Microsphere

The clinker/PVP autolytic mineral self-healing microspheres were prepared through several procedures. The main procedure was as follows: (1) the solution with 100 g cement clinker and 100 mL ethanol was stirred at 60 °C for 5 min. Then 3 g silane coupling agent KH-550 (except isothermal calorimetry test in Section 2.4.4) was added and the solution was stirred at 600 r/min for 30 min. After being kept for 1 hour, the pretreated clinker was obtained through filtering and drying. (2) A certain amount of PVP was dissolved in 300 mL ethanol and stirred to disperse for several minutes. Then, the solution was stirred for half an hour with the addition of pretreated clinker at 70 °C. Finally, the clinker/PVP autolytic microsphere was obtained by filtering and drying. During the preparation process, the mass ratio of PVP to clinker was changed to determine the thickness of the protective coating film shown in Table 2.

### 2.3. Mix Proportion and Specimen Preparation

The chemical composition of the type 525 ordinary Portland cement used is shown in Table 3. Cement paste with 0.35 w/c and dimensions of 25 × 25 × 25 mm was prepared. The replacement of clinker/PVP microsphere was 10%, 20% and 30% by mass of cement as shown in Table 4. The Mixing procedure was performed in accordance with Chinese standards (GBT 1346-2011). The hardened paste was demolded after 24 h and subsequently cured in a curing room (20 ± 2 °C and RH > 95%) until the testing day. At the age of 28 days, the specimens were pre-cracked using pre-compression to cause distributed microcracks [22]. The pre-compression load was held at 80% of the average ultimate resistance of three cubic specimens from the same batch. The total nine pre-cracked specimens of each content were placed in water with a temperature of 20 ± 2 °C until the following test day, and three pre-cracked specimens were used for each test age.

### 2.4. Test Procedure

#### 2.4.1. Morphology of Microsphere

The morphology of the microsphere was observed using an environmental scanning electron microscopy (ESEM, Quanta 200F) (FEI company, Oregon State, Hillsboro, USA) equipped with an energy dispersive spectrometer (EDS) (FEI company, Oregon State, Hillsboro, USA). The prepared autolytic microspheres were mixed into the cement paste, and the specimen was taken immediately for BSE-SEM observation after preliminary hardening.

#### 2.4.2. Chemical Structure of Microsphere

Fourier transform infrared spectroscopy (FTIR) (Bruker Optics, Karlsruhe, Germany) was used to determine the chemical structures of the clinker/PVP autolytic microsphere in order to identify the validity of film coating method.

#### 2.4.3. Coating Film Thickness

The autolytic microspheres were subjected to laser particle size analysis (LS320) (Beckman Coulter, California State, Brea, CA, USA). The average particle size was regarded as the diameter of the microspheres, and the formula for the coating film thickness is shown in Equation (1).

T = (D_m_ − D_0_)/2, (1)

where T is coating film thickness (μm), D_m_ is the particle diameter of microspheres (μm), and D_0_ is the particle diameter of cement clinker (μm).

#### 2.4.4. Autolytic Behavior

The autolytic time, defined as the time required for complete the autolysis of the coating film, is proposed to describe the autolytic behavior of microsphere [25]. The autolytic time is calculated as the time difference between the occurrence of two hydration exothermic heat peaks (reference and microsphere sample) according to equation (2). Therefore, the heat flow of paste was measured using isothermal calorimetry based on the Chinese standard GB12959-2008. These measurements of clinker and autolytic microsphere with 0 g, 1 g, 3 g, 5 g silane coupling agent (noted as SCA-0, SCA-1, SCA-3, SCA-5 respectively) were performed at 20 °C.

Tγ = Ta − Tr,(2)

where Tγ is the autolytic time (min), Ta is the time corresponding to the hydration exothermic peak of (clinker and water) paste (min), Tr is the time corresponding to the hydration exothermic peak of (microsphere and water) paste (min).

#### 2.4.5. Strength Recovery and Damage Degree of Self-Healing Specimens

A compressive strength test was performed to evaluate the self-healing effectiveness. The pre-cracked specimens were reloaded until failure to determine the strength recovery after water curing age of 2 days, 14 days and 28 days. The compressive strength recovery is determined by Equation (3).

γ = P_e_/P_c_, (3)

where γ is the compressive strength recovery (%), Pe is the ultimate compressive strength in reloading (MPa), Pc is the initial compressive strength (MPa).

The self-healing ability was also determined using the ultrasonic transmission method. Previous studies [31] showed that changes of ultrasonic properties can reflect the internal damage of cementitious materials, since the microstructure of materials will affect the ultrasonic transmission. Therefore, the damage degree was calculated according to Equation (4) to evaluate the recovery of internal closure, determined using an acoustic emission pulse test. The pulse transmission direction was vertical to the compression load direction. It is noted that the pulse test was conducted immediately after the pre-cracking of specimens and then carried out at the same day as the compressive strength test. The same specimens were used for both experiments, since the acoustic emission pulse test is a non-destructive experiment.

D = 1 − (T_0_/T_h_)^2^, (4)

where D is the damage degree, T_0_ is the pulse time of specimen before pre-compression, Th is the pulse time of specimen after self-healing.

## 3. Results

### 3.1. Characterization of Clinker/PVP Microsphere

#### 3.1.1. Morphology and Microstructure Analysis

**Figure 1:**
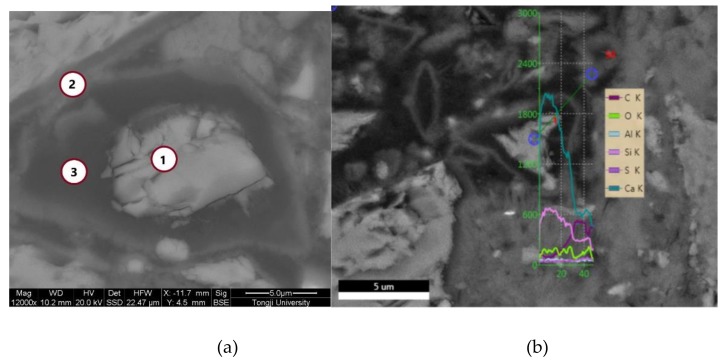
Micro-morphology of autolytic microspheres by BSE-SEM (**a**) Spot analysis; (**b**) Line analysis.

The prepared autolytic microsphere was mixed into the cement paste and the backscattering specimen was prepared after preliminary hardening. The results of BSE-SEM observation are shown in Figure 1.

**Figure 2:**
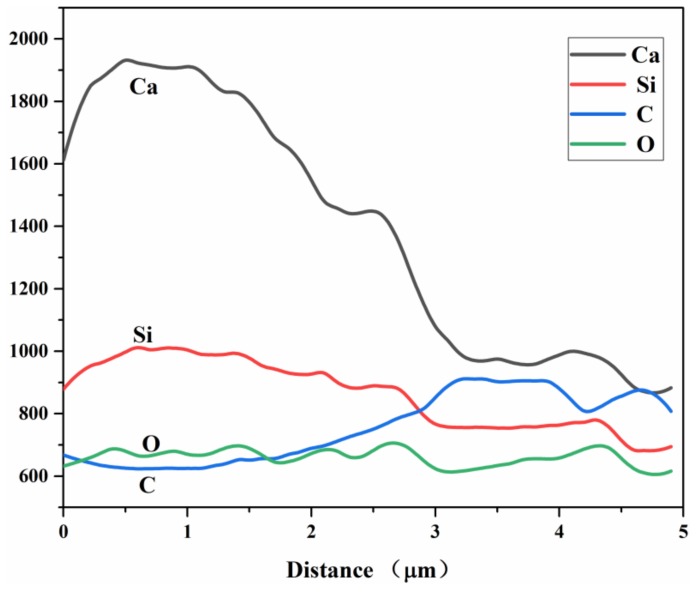
Energy dispersive spectrometer (EDS) line analysis results of autolytic microspheres.

Based on the BSE-SEM imaging principle, the BSE-SEM imaging brightness is determined by the average atomic number. The region with a higher average atomic number presents a stronger backscattered electron signal and exhibits a brighter gray scale in the BSE images [32]. The results in Figure 1(a) show that the whole autolytic microsphere presents irregular shape. This is mainly due to the shape of the clinker. The brightest material in the middle is the remaining cement clinker, which is the healing agent. To further confirm the dark materials are PVP film, the chemical composition measured by spot and line analysis of the EDS are shown in Table 4 and Figure 2, respectively.

The results in Table 5 show that point 1 is cement clinker particles with a mass ratio of calcium to silicon equaling to 4.39. Point 2 is the boundary between the darkest material edge and the surrounding hydration product, the mass ratio of carbon element increases while the mass of calcium silicon ratio decreased to 4.14. In addition, mass ratio of the carbon element takes up more than 60% in Point 3 while the mass ratio of the oxygen element also improves significantly compared with other two positions. Therefore, the dark region is indeed the PVP film layer due to the chemical composition of PVP mainly consisting of carbon and oxygen elements. The results shown in Figure 2 show that the relative content of Ca and Si elements in the position of the protective film layer from the autolytic microspheres to the marginal PVP film are decreasing in sequence, while the relative content of C and O elements are increasing in sequence. The results also prove that the protective effect of PVP protection film can be achieved.

**Figure 3:**
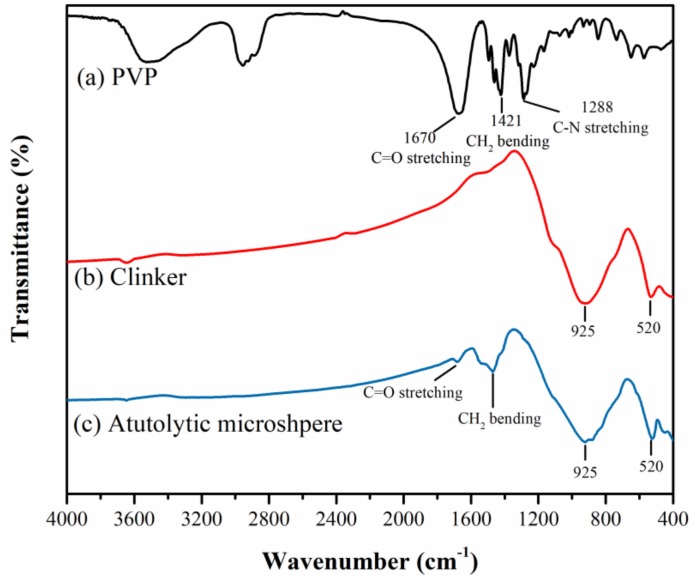
Fourier transform infrared spectroscopy (FTIR) spectra of (**a**) PVP (polyvinyl pyrrolidone), (**b**) clinker, (**c**) clinker/PVP autolytic microsphere.

Figure 3 shows the FTIR spectra results of the only PVP system, the only clinker system and the clinker/PVP autolytic microsphere system. In the present study [33,34], the IR band at 1288 cm^−1^ and 1421 cm^−1^ of the only PVP system are assignable to C-N stretching and CH_2_ bending vibration, respectively. The strong C=O stretching vibration at 1670 cm^−1^ is also observed, which is the most significant characteristic vibration of PVP. The strong vibration of the only clinker system at 520 cm^−1^ and 925 cm^−1^ may correspond to the out-of-plane bending and anti-symmetrical stretching of SiO_4_ tetrahedra [35]. With respect to the spectra of the autolytic microsphere, strong bands—at 1670 cm^−1^ and 1421 cm^−1^—are observed. This may explain that clinker was successfully coated with PVP film. Furthermore, the characteristic vibration of clinker at 925 cm^−1^ and 520 cm^−1^ can be clearly found in autolytic microsphere system. This confirms that clinker remains the original mineral healing composition with the coating of PVP film on the clinker surface.

#### 3.1.2. Coating Film Thickness and Size Distribution

**Figure 4:**
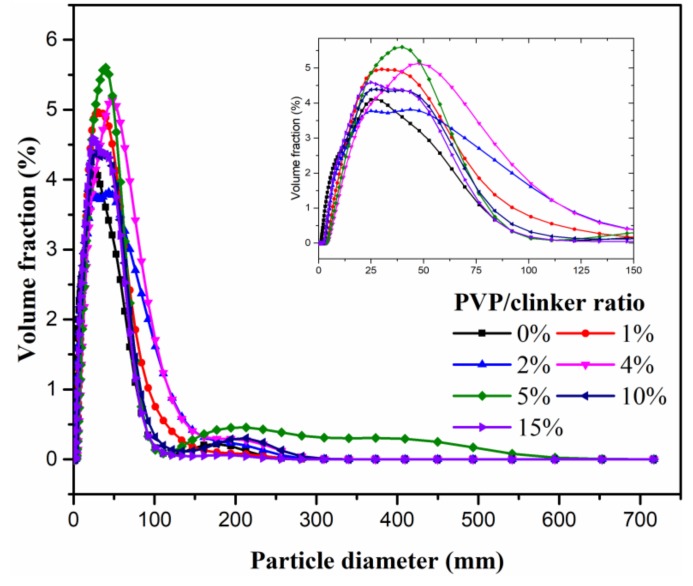
Particle size distribution of autolytic microspheres with different PVP/clinker ratio.

The particle size distribution of the clinker/PVP microsphere is shown in Figure 4. The results in Figure 4 show that the particle size first shifts to the larger ones as the PVP/clinker ratio increases from 0% to 4%, and then the size shifts to the smaller ones as the PVP/clinker ratio increases to 15%. This is mainly due to the increase in the viscosity of the stirring solution with the increasing amount of coating materials. The dispersion degree of the solution decreases once the critical amount is exceeded. In addition, all autolytic microspheres present a similar size distribution to clinker, each having sizes on the order of tens of microns. This means that addition of the clinker/PVP autolytic microsphere will not affect the particle accumulation of the ordinary matrix. This is an outstanding advantage when cement is replaced with autolytic microsphere. Based on the particle size distribution results, the average film thickness is calculated as shown in Table 6. The maximum thickness of coating film reaches at 7.54 μm in the PVP/clinker ratio of 4%.

#### 3.1.3. Autolytic Behavior Analysis

**Figure 5:**
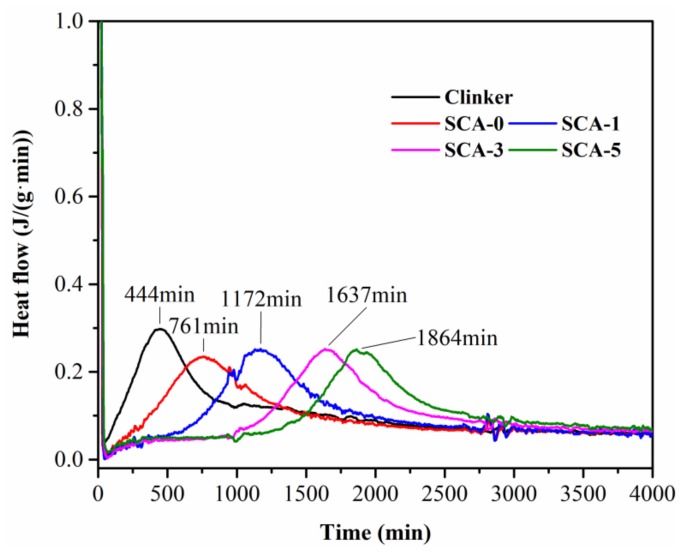
Heat evolution of clinker and microsphere paste with w/c ratio of 0.35 measured by isothermal calorimetry.

The control of autolytic behavior is important to avoid the premature consumption of microsphere which means enough autolytic time is needed [25]. Thus, the heat evolution of the clinker and autolytic microsphere paste were determined, and the results are shown in Figure 5. The hydration exothermic peak time of clinker appears at 444min. The autolytic time of clinker/PVP microspheres with 0 g, 1 g, 3 g and 5 g silane coupling agent are calculated as 317 min, 728 min, 1193 min and 1420 min according to equation (2), respectively. In terms of autolytic time, the clinker without pretreatment (i.e., with 0 g silane coupling agent) is much less than that of the clinker with pretreatment. The autolytic time of clinker with only 1g silane coupling agent presents 130% higher than that of SCA-0 sample, which means the pretreatment process is necessary to control the autolytic behavior of the clinker/PVP autolytic microsphere. In addition, autolytic time of microspheres get higher with increase in silane coupling agent amount. Experimental results show that the autolytic time can be controlled by the preparation process to meet different engineering requirements.

### 3.2. Self-Healing Effectiveness of Specimen with Microsphere Prepared

#### 3.2.1. Recovery of Compressive Strength

**Figure 6:**
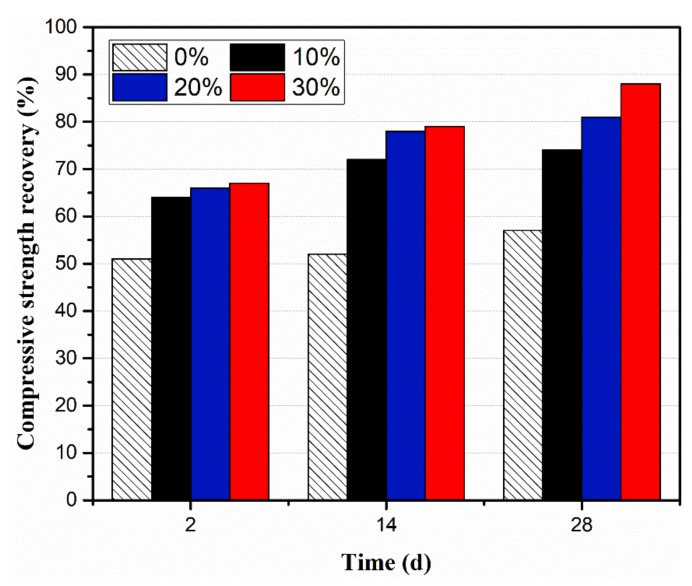
Compressive strength recovery at different curing time.

The compressive strength is the main basis for quality control in civil engineering. Thus, compressive strength recovery is chosen as one of the evaluation methods for self-healing effectiveness. The clinker/PVP autolytic microsphere with a 7.54 μm film thickness was adopted in the cement paste specimen. The compressive strength recovery of the specimen at 2 days, 14 days and 28 days after pre-crack is shown in Figure 6.

Results show that the compressive strength recovery of all the specimen-incorporating microspheres were higher than ordinary group at 2 days, 14 days and 28 days. The ordinary group of specimens—those not incorporating autolytic microspheres—have limited self-healing potential in terms of 51% compressive strength recovery at 2 days, and only 6% improvement from 2 days to 28 days. The specimens incorporating microspheres show more obvious self-healing ability. The compressive strength recovery rates of those specimens with microspheres were in the range of 64%–67%, 72%–79%, and 74%–88% at 2 days, 14 days and 28 days, respectively. Moreover, the compressive strength recovery of specimens with 30% microspheres shows more improvement from 2 days to 28 days compared with the left two groups. Concerning the healing mechanism [19,36], this is due to more unhydrated clinker provided by clinker/PVP autolytic microsphere. On the other hand, compressive strength recovery of specimens with a 30% microsphere reaches the maximum of 88% at the age of 28 days, which is 54% higher than that of ordinary cement paste specimens. Ongoing hydration is conducive to crack healing in autogenous self-healing cementitious materials. Experimental results confirm that self-healing properties is enhanced due to higher unhydrated clinker encouraged by the clinker/PVP microsphere.

#### 3.2.2. Recovery of Damage Degree

**Figure 7:**
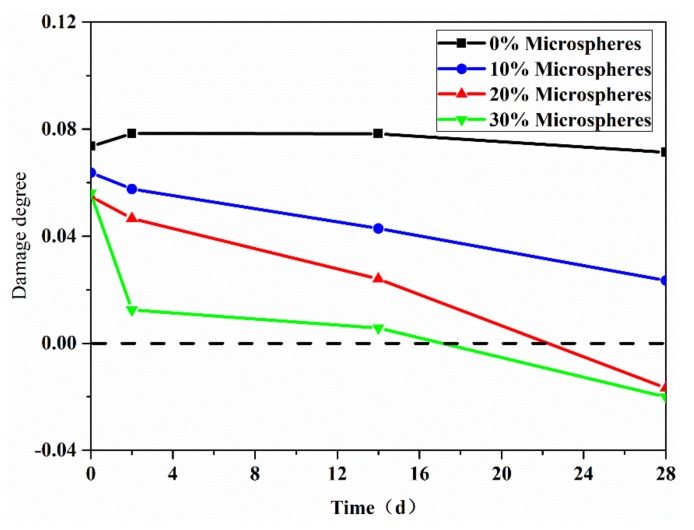
Effect of microsphere amount on damage degree.

To evaluate the internal closure recovery, damage degree was measured based on the ultrasound pulse time [37]. The damage degree will decrease once the internal crack closure is achieved. The effect of microsphere amount on damage degree is shown in Figure 7. Results show that the damage degree of the specimens with the microsphere decreased on elapsed time compared with the ordinary group. The damage degree of ordinary kept almost constant for 28 days. This means there was no significant internal crack healing in this group, which is consistent with the compressive strength recovery results. The damage degree of specimens with autolytic microspheres experienced a significant decrease, especially in those groups with 20% and 30% microspheres at the age of 28 days. These two groups even fell below the zero-standard line, which may be due to further microcrack healing besides the crack induced by pre-compression. Combined with the results of compressive strength recovery, the healing effectiveness may have more effect on permeability rather than on compressive strength.

## 4. Conclusions

In this paper, a clinker/PVP autolytic microsphere is proposed. A study on the preparation and characterization of autolytic microspheres is presented. The self-healing effectiveness of specimens with autolytic microspheres is also evaluated. Several conclusions can be obtained from the experimental results:

- The clinker/PVP autolytic microsphere is successfully prepared, and the self-healing potential is confirmed via the chemical structure measured by FTIR.
- The film coating thickness increases first and then decreases with the increasing usage of coating materials, since there exists the optimal viscosity of solution. The maximum film thickness was 7.54 μm in the PVP/clinker ratio of 4%.
- The autolytic behavior of the clinker/PVP autolytic microsphere is successfully controlled by pretreatment degree (i.e., silane coupling agent amount).
- The self-healing potential of specimens with autolytic microspheres improves with an increase in the microsphere amount.
- The self-healing effectiveness of clinker/PVP autolytic microspheres is confirmed by the compressive strength recovery and damage degree tests.

## Figures and Tables

**Table 1:** Chemical composition of cement clinker.

CaO	SiO_2_	Fe_2_O_3_	Al_2_O_3_	MgO	SO_3_	Na_2_O	K_2_O	Other
63.1	24.1	3.27	4.34	1.16	0.76	0.12	0.8	2.35

**Table 2:** Proportion used for preparation of coating film.

Description	No. 1	No. 2	No. 3	No. 4	No. 5	No. 6	No. 7
PVP/clinker ratio	0%	1%	2%	4%	5%	10%	15%

**Table 3:** Chemical composition of Portland cement.

Component	SiO_2_	Al_2_O_3_	Fe_2_O_3_	CaO	MgO	TiO_2_	SO_3_	Na_2_O	K_2_O	LOI
Content (wt.%)	20.0	4.51	3.11	64.3	0.68	0.22	2.99	0.03	0.72	2.70

**Table 4:** Mixture proportion for test.

Description	w/c	Microsphere Fraction (wt. % with Cement)	Specimens Number	Test Method
Compression	0.35	0	3	For ultimate resistance
	0.35	10	3	
	0.35	20	3	
	0.35	30	3	
Pre-compression	0.35	0	9	For strength recovery and pulse test
	0.35	10	9	
	0.35	20	9	
	0.35	30	9	

**Table 5:** Mass Distribution of Major Elements in Different Regions.

Element Weight %	Ca	Si	C	O
Point 1	37.01	8.44	11.43	43.12
Point 2	17.07	4.12	45.08	33.14
Point 3	9.69	2.34	62.27	25.07

**Table 6:** The mean diameter and average film thickness of autolytic microspheres.

Description	No. 1	No. 2	No. 3	No. 4	No. 5	No. 6	No. 7
PVP/clinker ratio	0%	1%	2%	4%	5%	10%	15%
Mean diameter (μm)	21.05	27.20	27.50	36.12	32.54	26.16	24.62
Average film thickness (μm)	0	3.08	3.23	7.54	5.75	2.56	1.79
